# Dendritic Ca^2+^ dynamics and multimodal processing in a cricket antennal interneuron

**DOI:** 10.1152/jn.00663.2017

**Published:** 2018-05-09

**Authors:** Timothy George Bayley, Berthold Hedwig

**Affiliations:** Department of Zoology, University of Cambridge, Cambridge, United Kingdom

**Keywords:** Ca^2+^ imaging, dendritic processing, multimodal stimulation

## Abstract

The integration of stimuli of different modalities is fundamental to information processing within the nervous system. A descending interneuron in the cricket brain, with prominent dendrites in the deutocerebrum, receives input from three sensory modalities: touch of the antennal flagellum, strain of the antennal base, and visual stimulation. Using calcium imaging, we demonstrate that each modality drives a Ca^2+^ increase in a different dendritic region. Moreover, touch of the flagellum is represented in a topographic map along the neuron’s dendrites. Using intracellular recording, we investigated the effects of Ca^2+^ on spike shape through the application of the Ca^2+^ channel antagonist Cd^2+^ and identified probable Ca^2+^-dependent K^+^ currents.

**NEW & NOTEWORTHY** Different dendritic regions of the cricket brain neuron DBNi1-2 showed localized Ca^2+^ increases when three modalities of stimulation (touch of the flagellum, strain at antennal base, and visual input) were given. Touch stimulation induces localized Ca^2+^ increases according to a topographic map of the antenna. Ca^2+^ appears to activate K^+^ currents in DBNi1-2.

## INTRODUCTION

Multimodal processing is essential to adjust the output of the nervous system given a range of sensory inputs. In the cricket brain, a giant interneuron, DBNi1-2 (descending brain neuron ipsilateral 1-2), responds to three modalities of input: touch to the flagellum (the elongate region comprising the majority of the antenna), strain of the antennal base, and visual stimulation (Gebhardt and Honegger 2001; Schöneich et al. 2011; Staudacher and Schildberger 2000). In this study, we investigated how the location of its synaptic inputs, and morphology of its dendrites, contributes to multimodal processing.

The overlap between DBNi1-2 dendrites and projections of neurons carrying sensory information differs for each modality. Hairlike touch receptors, trichoid sensilla (Pflüger 1980; Pumphrey 1936), are located along the flagellum (Fudalewicz-Niemczyk and Rościszewska 1973; Staudacher et al. 2005; Watanabe et al. 2012). Their axonal terminals overlap with large “fingerlike” dendrites of DBNi1-2 (Gebhardt and Honegger 2001; Staudacher and Schildberger 2000). Strain receptors, campaniform sensilla (Pringle 1938a, 1938b; Spinola and Chapman 1975), are found at the base of the antenna (Fudalewicz-Niemczyk and Rościszewska 1973; Staudacher et al. 2005). Their terminals overlap with a different region, the junction between the fingerlike dendrites and the axon descending to the thoracic segments (Schöneich 2008). Visual input to DBNi1-2 comes via the optic stalk (Gebhardt and Honegger 2001), and projections from it overlap with a more anterior dendritic region (Gebhardt and Honegger 2001; Honegger and Schürmann 1975; Schöneich et al. 2011). These morphological features imply that the sensory input of each modality is received in a separate dendritic region of DBNi1-2. Sensory responses of the neuron may be reduced during active antennal movements (Gebhardt and Honegger 2001), but this is not addressed in the present study.

The spatial arrangement of synaptic inputs to a neuron can be revealed using in vivo optical imaging techniques. In the present study, we focused on Ca^2+^ imaging, because it has been studied extensively and indicators are reliable. Considerable progress has been made through single-cell calcium imaging of large neurons in insects, where the entire range of sensory afferents of a particular modality forms connections with a single neuron, such as for mechanosensory (Ogawa et al. 2004), auditory (Baden and Hedwig 2007; Prešern et al. 2015), and visual interneurons (Borst and Egelhaaf 1992; Hopp et al. 2014; Peron et al. 2009). The location of synaptic inputs to single neurons has also been studied in mammals, including amacrine cells in the retina (Euler et al. 2002; Grimes et al. 2010) and various brain neurons, such as pyramidal neurons in the cortex (Jia et al. 2010) and hippocampus (Sheffield and Dombeck 2015). However, in vertebrates, each neuron often only receives a subset of the afferent input of a sensory field.

Although the role of DBNi1-2 in behavior is not yet known, the spatial separation of sensory inputs has important functional implications for how multiple inputs are processed within a single neuron (reviews: Koch and Segev 2000; London and Häusser 2005). We have investigated whether different dendritic regions of DBNi1-2 show a Ca^2+^ increase in response to touch and strain of the antenna and to visual stimulation. By combining Ca^2+^ imaging with intracellular recordings, we first assess the restriction of Ca^2+^ signals to specific dendritic regions. Second, we explore the role of Ca^2+^ on neuronal processing through the application of the Ca^2+^ channel blocker Cd^2+^.

## MATERIALS AND METHODS

### 

#### Animals.

Adult male and female crickets (*Gryllus bimaculatus*) were taken from a stock in the Department of Zoology. Crickets were kept on a 12:12-h light-dark cycle and fed ad libitum with cat food, fish flakes, muesli, and water.

#### Dissection.

**Fig. 1.**
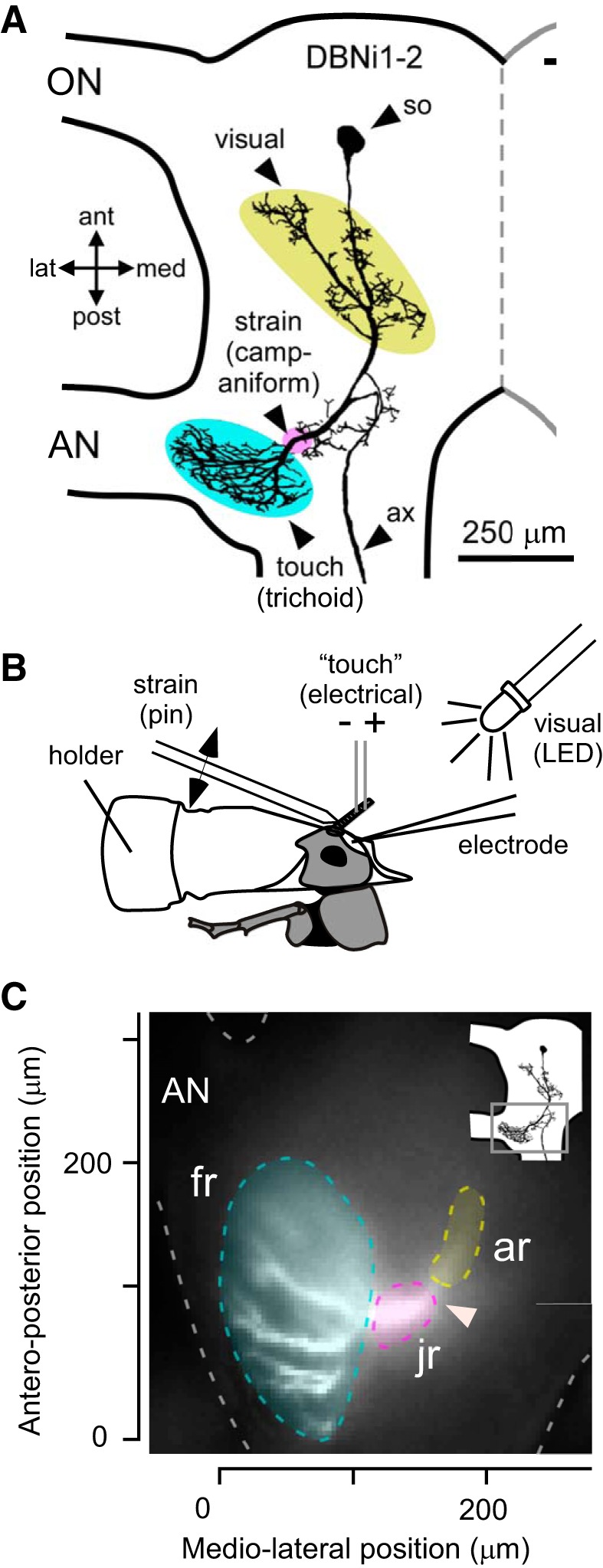
Neuronal morphology and experimental overview. *A*: outline of half of the brain with the optic nerve (ON) and the antennal nerve (AN). The morphology of DBNi1-2 is reconstructed from an Alexa 568 staining, with the soma (so) and descending axon (ax) indicated. Areas shaded in color show putative dendritic regions receiving input from touch-sensitive trichoid sensilla (cyan), strain-sensitive campaniform sensilla (magenta), and visual neurons (yellow). ant, Anterior; lat, lateral; med, medial; post, posterior. *B*: experimental setup showing holder for the head made from a modified Eppendorf tube, as well as stimulation methods: electrical stimulation of touch receptors, mechanical stimulation of strain-sensitive receptors with a pin at the base of the antenna, and visual stimulation with a white light-emitting diode (LED). *C*: average of 50 frames of fluorescence signal during in vivo imaging with an outline of the brain (dashed gray line) and the AN. Regions shaded in color regions indicate the fingerlike dendritic region (fr), the junction region of these projections (jr), and a separate anterior dendritic region (ar). *Inset* brain diagram (*top right*) shows region imaged (gray square). Axes indicate calibration between animals and represent the distance from the posteriormost position (origin of *y*-axis) and lateralmost position (origin of *x*-axis) of the dendritic region.

The head was secured in a modified Eppendorf tube after the mouthparts were removed (Fig. 1*B*). The tube was filled with saline of ionic concentrations (in mM): 135 NaCl, 10 KCl, 7 CaCl_2_, 8 NaHCO_3_, 1 MgCl_2_, 4.8 TES, and 4.4 trehalose. The brain was then exposed, with the descending connectives left intact but with antennal motor nerves N2, N3, and N4 cut (Honegger et al. 1990). The brain was supported on a stainless steel platform, attached to a metal tube through which a light guide was passed to illuminate the recording site.

#### Electrophysiology.

For intracellular recording, borosilicate glass capillaries (outer diameter, 1.0 mm; inner diameter 0.7 mm; Hilgenberg, Malsfeld, Germany) were pulled using a DMZ Universal Puller (Zeitz Instruments, Martinsried, Germany) to form electrodes with resistance of 50–70 MΩ when filled. For imaging, two dyes were introduced to the microcapillaries by backfilling: a Ca^2+^ indicator, 0.5 mM Oregon green 488 BAPTA-1 (OGB-1; Molecular Probes, Life Technologies, Paisley, UK), and a fixable dye that allowed the morphology of the neuron to be inspected after imaging, 0.15 mM Alexa Fluor-568 (Alexa-568; Molecular Probes). Dyes were dissolved in UltraPure water (Invitrogen, Life Technologies, Paisley, UK) to ensure a low Ca^2+^ concentration. The shaft was filled with 1 M K-acetate. The brain was viewed using a DM-LFS microscope (Leica Microsystems, Wetzlar, Germany) equipped with ×4 and ×10 air objectives and ×10 and ×20 water-immersion objectives. The focal neuron, DBNi1-2, was located visually from landmarks on the surface of the brain, with the electrode inserted at the junction between the antennal lobe and ventral area of flagellar afferents (Staudacher and Schildberger 2000). It was further characterized electrophysiologically from its large background level of excitatory postsynaptic potentials, as well as its response to light and antennal touch, which is very uncommon for neurons within this region of the brain (Gebhardt and Honegger 2001; Schöneich et al. 2011). The electrode signal was amplified with an SEC-10LX amplifier (npi electronic, Tamm, Germany), fed into a PowerLab 8/30 data acquisition system (AD Instruments, Oxford, UK), and recorded at 50 kHz using LabChart software (AD Instruments). For morphological analysis, the brain was dissected, fixed in 5% paraformaldehyde, dehydrated for 30 min each in a series of 70, 90, 95, and 100% ethanol, and cleared in methyl salicylate. After clearing, images were taken either with an EOS 350D digital single-lens reflex camera (Canon UK, Reigate, UK) mounted on an Axiophot microscope (Carl Zeiss Microscopy, Cambridge, UK) or with a TCS-SP-5 confocal microscope (Leica), using LAS AF software.

#### Optical imaging.

An Optoscan monochromator (Cairn Research, Kent, UK) provided a uniform epifluorescent illumination of the preparation with light of 488 ± 15 nm to excite OGB-1 or 568 ± 15 nm to excite Alexa-568. Light emitted by the indicator was captured by an iXon DV887DCS-BV cooled charge-coupled device camera (Andor Technology, Belfast, UK) mounted onto the microscope. Images (128 × 128 pixels) were acquired at 50 Hz, with 20-ms exposure time, using Andor iQ software. For synchronization with electrophysiological data, the camera provided pulses when each frame was captured, which were recorded simultaneously with electrophysiological data. Fluorescence signals are expressed as the relative percentage of change in fluorescence from background (ΔF/F).

**Fig. 2.**
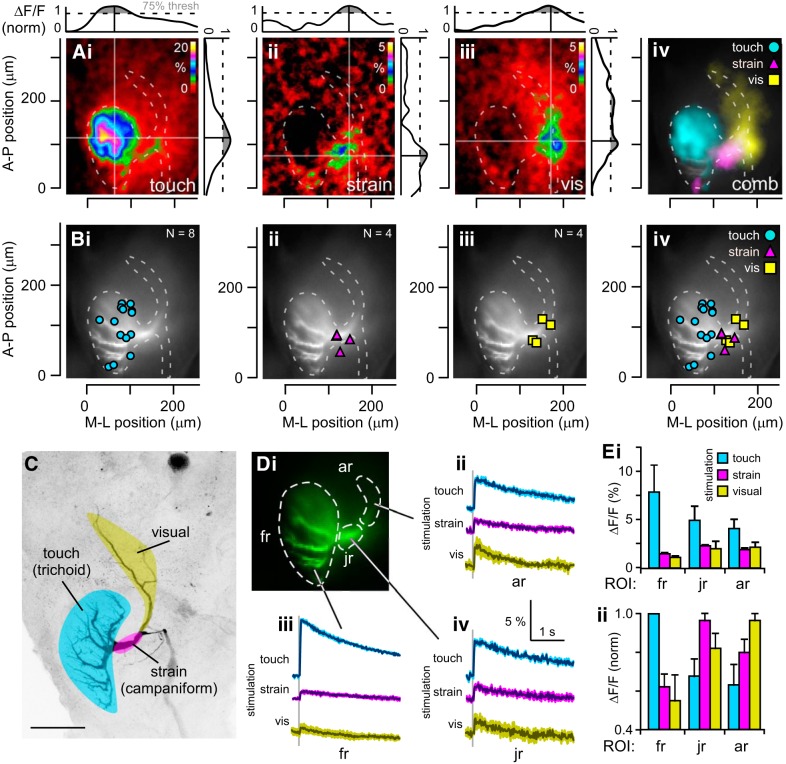
Locating functional input sites to DBNi1-2. *Ai–Aiii*: representative example of the midpoint of the Ca^2+^ response to touch receptor stimulation (*i*), strain receptor stimulation (*ii*), and visual stimulation (vis; *iii*), each based on 30–40 repeats per stimulus, recorded in one animal. Values for the relative change in fluorescence (ΔF/F, %) were binned over 2.5 pixels in *x* and *y*, and over 2.5 frames in time. Plots next to each image show the fluorescence profile along the mediolateral (M-L) axis (*top*) and the anteroposterior (A-P) axis (*right*); values are normalized (norm) to the pixel with highest fluorescence change. Shaded regions indicate pixel values over the 75% threshold, used for calculating the midpoint of the fluorescence response (see materials and methods). The midpoint is indicated with black lines in plots and gray lines in central images. Axes next to images *Ai–Aiv* scale distance along the A-P dimension (*left*) and the M-L dimension (*bottom*). The origin of the axes is defined by the posteriormost point and the lateralmost point of the fingerlike dendrites; posterior is to the bottom and lateral to the left of the images. *Aiv*: combined imaging responses for all touch, strain, and visual stimulation tests, with color code for sensory modalities at *top right*. *Bi–Biv*: midpoints of fluorescence responses to touch stimulation (*i*), strain receptor activation (*ii*), and visual stimulation (*iii*), and overlay of all the midpoints in response to each modality of stimulation (*iv*). *N* = no. of preparations; note the complete overlap of 2 data points for strain. *C*: a schematic, simplified representation of sensory input regions as indicated by Ca^2+^ increases based on plots in *Aiv* and *Biv* is overlaid with the maximum projection of a confocal stack from DBNi1-2 as revealed by staining with Alexa 568; data are from the same animal. Scale bar, 100 μm. *Di*: fluorescence responses over time obtained from 3 different regions of interest (ROIs), i.e., fingerlike dendrites (fr), anterior region (ar), and junction region (jr), corresponding to the sensory input sites. *Dii–Div*: responses to touch (cyan), strain receptor activation (magenta), and visual stimulation (yellow) are shown for ROI ar (*ii*), fr (*iii*), and jr (*iv*). Stimulus onset is indicated by vertical gray line; data are averages of 5–8 presentations per stimulus. All recordings are from one animal, different from animal in *A*. *E*: quantification of imaging responses. Peak fluorescence response is shown as ΔF/F (%) for each ROI (fr, jr, and ar) for touch stimulation, strain receptor activation, and visual stimulation (*i*), and peak responses for each sensory modality are normalized to the ROI showing the highest response (*ii*). *N* = 4 preparations.

To quantify the position of Ca^2+^ increase in the neuron, the midpoints of the fluorescence profiles in the anteroposterior (A-P) and mediolateral (M-L) axes were derived. These are based on calculations for the center of mass of an object (e.g., Fig. 2*Ai*). For a rectangular region of interest (ROI), the mean ΔF/F along the M-L axis was calculated by averaging the values of all corresponding pixels in the A-P axis: all A-P data were collapsed on the M-L axis, e.g., pixel (M-L_1_/A-P_1_) is the average value calculated considering the pixels (M-L_1_/A-P_1_) to (M-L_1_/A-P*_n_*), etc. The mean ΔF/F along the A-P axis was calculated correspondingly by averaging the values of all pixels in the M-L axis: all M-L data were collapsed on the A-P axis, e.g., pixel (M-L_1_/A-P_1_) is the average value calculated considering the pixels (M-L_1_/A-P_1_) to (M-L*_n_*/A-P_1_). These values were smoothed with a 10-pixel moving average before the pixels above 75% of the maximum ΔF/F intensity were selected, and all pixels were normalized to a value of 0–1. For pixels above 75%, the distance of each pixel from the posteriormost or lateralmost position was multiplied by its normalized intensity and divided by the sum of the normalized intensity of all the pixels above 75% intensity. This provided the midpoint of fluorescence in the A-P and M-L axis, respectively. Anterior and posterior were defined according to the neuraxis of insect nervous systems (Ito et al. 2014).

#### Stimulation.

On the basis of morphological data, the descending antennal interneuron DBNi1-2 is thought to receive inputs in distinct dendritic regions from sensory neurons responding to touch of the antennal flagellum (Gebhardt and Honegger 2001; Staudacher and Schildberger 2000), strain at the base of the antenna (Schöneich 2008), or visual stimulation of the compound eyes (see Fig. 1*A*; Gebhardt and Honegger 2001; Honegger and Schürmann 1975; Schöneich et al. 2011). For touch-sensitive trichoid sensilla (Staudacher and Schildberger 2000) and strain-sensitive campaniform sensilla (Schöneich 2008), simultaneous labeling of the sensory fibers and DBNi1-2 revealed a close spatial association between the afferent axons and the dendrites of DBNi1-2. For visual input, stainings were not obtained in the same animals (Gebhardt and Honegger 2001; Honegger and Schürmann 1975).

To activate touch-sensitive mechanoreceptors on the flagellum, electrical stimulation was used, because mechanical stimulation of the flagellum also activates the campaniform sensilla at the antennal base (Schöneich 2008). The afferent fibers are thought to project in the flagellar nerve such that mechanoreceptors at the tip of the flagellum run through the center of the nerve and those further toward the base run more laterally (Fudalewicz-Niemczyk and Rościszewska 1973). To ensure only the mechanoreceptors at the focal site were activated, small stimulus intensities were used to excite only more lateral afferents in the nerve. Up to three small incisions were made in the flagellar cuticle, into which two 20-μm steel wires (Rheinische Feindraht Industrie, Eckenhagen, Germany) were inserted, insulated up to their tips. The area was coated with petroleum jelly to prevent desiccation. Stimulation was with a 1-ms pulse of 1-V amplitude, which would drive one or two spikes in DBNi1-2. Further details are given later in the text.

To excite strain-sensitive campaniform sensilla at the base of the antenna, 2.5 mN tactile stimuli were presented with an insect pin glued to a small speaker cone. The base of the antenna is also endowed with several other receptor neuron types, including the sensilla of the scapal hair plate and a chordotonal organ, as well as trichoid and basiconic sensilla (Fudalewicz-Niemczyk and Rościszewska 1973; Rospars 1988). The receptor neurons from the scapal hair plate and chordotonal organ pass through a separate nerve branch, different from the other receptor types (Gebhardt and Honegger 2001). Previous experiments have shown that stimulating this branch does not induce a response in DBNi1-2, suggesting that there is no synaptic input from these sensory neurons (Gebhardt and Honegger 2001). The chemosensory sensilla terminate in a separate neuropile, which does not overlap with the dendrites of DBNi1-2 (Staudacher and Schildberger 2000). Therefore, inputs to DBNi1-2 should primarily arise from the trichoid sensilla, projecting to the fingerlike dendrites, and from campaniform sensilla, projecting to the junction region of these dendrites (Schöneich 2008).

Visual stimuli were presented using a white light-emitting diode (LED) placed 15 cm from the right eye of the cricket. Light pulses were of 10-ms duration and were driven by scripts written in Spike2 (CED, Cambridge, UK). Exact stimulus parameters are provided in the text. The stimuli may have activated both the compound eyes and ocelli; however, input to DBNi1-2 is likely to come only from the eyes: first, when the optic stalks are ablated, the visual input to DBNi1-2 is abolished (Gebhardt and Honegger 2001); and second, the dendrites of DBNi1-2 overlap with the neurons projecting from the optic stalk (Honegger and Schürmann 1975) and not with those from the ocelli (Koontz 1976). Epifluorescent illumination also stimulated the visual system, but spiking subsided within 1 s.

#### Blockade of Ca^2+^ channels.

To block Ca^2+^ channels, saline containing 0.1 M CdCl_2_ was continually passed over the preparation. Excess saline was removed with a Masterflex L/S pump (Cole-Palmer Instrument, London, UK).

#### Data analysis.

Data were processed with Spike2 (CED) and Excel (Microsoft, Redmond, WA), with statistical tests carried out using R (R Foundation, Vienna, Austria). Images were analyzed using ImageJ, Spike2, and Python 2.7 (Python Software Foundation, Wilmington, DE). Data were tested for normality with a Shapiro-Wilk test. Values given in the text are means ± SE unless otherwise stated.

## RESULTS

To identify whether the different sensory modalities activate different dendritic regions of DBNi1-2, the Ca^2+^ indicator OGB-1 was introduced through a glass microelectrode, and each modality was presented independently (Fig. 1, *A* and *B*). DBNi1-2 was imaged in the brain using epifluorescence microscopy. This technique allows large sections of the neuron to be imaged simultaneously, including a fingerlike region of dendritic arborization (Fig. 1*C*, “fr”), the junction region of these dendrites (Fig. 1*C*, “jr”), and a separate anterior region (Fig. 1*C*, “ar”), representing a section of the projection to the putative site of visual input. The more anterior dendritic regions are located dorsally, deep within the brain, and because of the resulting light scatter could not be imaged clearly. The accessible regions, however, were imaged at high spatial and temporal resolution. The site of Ca^2+^ increase was calibrated between animals by aligning to the posteriormost and lateralmost position of the neuron (Fig. 1*C*).

### 

#### Modality-specific localization of Ca^2+^.

A modality-specific localization of Ca^2+^ signals should only occur if *1*) synaptic inputs are spatially separated on the basis of modality; *2*) a Ca^2+^ increase occurs at a dendrite when it receives synaptic inputs; and *3*) any such Ca^2+^ increase is restricted to a specific region of the dendrites. Spiking activity may drive Ca^2+^ increases, which would be expected to be highest in the spike-generating zone (Peron and Gabbiani 2009). In DBNi1-2, this is likely to be where the dendritic regions converge (Gwilliam and Burrows 1980) and where the descending axon originates (Fig. 1*C*, arrowhead). If Ca^2+^ influx is derived from spikes, it would be expected to occur in this same position for all modalities of input.

To quantify the location of Ca^2+^ increases in the DBNi1-2 dendrites during sensory stimulation, the fluorescence profile was measured from the start of stimulation for 400 ms (or 20 frames) for the M-L and A-O axes (see profiles at *top* and *right* of Fig. 2, *Ai–Aiii*, respectively; see materials and methods). A threshold was taken at 75% (shaded region in profiles), and the weighted midpoint of the pixels above the threshold was then calculated (lines in Fig. 2, *Ai–Aiii*; see materials and methods). The range and mean position of the midpoint were calculated between animals. Data are from four animals responding to all three stimuli, with a further four analyzed for their response to trichoid sensilla.

When touch-sensitive trichoid sensilla were electrically stimulated, in the example shown, the fluorescence midpoint occurred 61 μm medially from the lateralmost point of the neuron and 113 μm anteriorly from the posteriormost point (Fig. 2*Ai* and Supplemental Movie S1, available in the data supplement online at the *Journal of Neurophysiology* website). Across all the preparations, stimulated at various points along the flagellum, it fell between 37 and 112 μm medially and between 16 and 158 μm anteriorly, with a mean position of 87 ± 7 μm medially and 104 ± 13 μm anteriorly (*N* = 8; Fig. 2*Bi*). In all animals, therefore, the fluorescence midpoints fell within the area of the fingerlike dendrites, onto which flagellar mechanoreceptors are thought to form synapses (Staudacher and Schildberger 2000).

When strain-sensitive campaniform sensilla at the antennal base were activated in the animal in the example, the fluorescence midpoint was different, occurring 143 μm medially and 74 μm anteriorly (Fig. 2*Aii* and Supplemental Movie S1). Between preparations, it occurred from 124 to 143 μm medially and 51 to 86 μm anteriorly, with a mean position of 129 ± 3 μm medially and 74 ± 8 μm anteriorly (*N* = 4; Fig. 2, *Aii* and *Bii*). These points are all around the junction region of the fingerlike dendrites (note that 2 data points completely overlap), where campaniform sensilla from the base of the antenna have been indicated to form synapses with DBNi1-2 (Schöneich 2008).

Stimulating the visual system with light pulses (Fig. 2*Aiii* and Supplemental Movie S1) drove a fluorescence increase with its midpoint 168 μm medially and 105 μm anteriorly. Between preparations, the position of the strongest response ranged from 133 and 176 μm medially and 68 and 112 μm anteriorly, with a mean position of 151 ± 9 μm medially and 88 ± 11 μm anteriorly (*N* = 4; Fig. 2*Biii*). These points fall within the junction region of the fingerlike dendrites and the neurite projecting to the anterior dendrite, which is indicated to overlap with visual neurons (Gebhardt and Honegger 2001; Honegger and Schürmann 1975; Schöneich et al. 2011).

When the fluorescence images for all three stimulus modalities were overlaid, the spatial distribution of the responses showed little overlap (Fig. 2*Aiv*). Similarly, when the midpoints to all modalities of stimulation were overlaid (Fig. 2*Biv*), the regions activated by flagellar stimulation were distinct from the regions activated by other stimuli. Only the regions active upon antennal base stimulation and visual stimulation showed an overlap. This overlap is likely to be accentuated by scatter of the fluorescent light in the tissue, which will reduce and broaden the signal from the deep anterior dendrites receiving visual inputs. Spike-derived Ca^2+^ influx may also contribute to blurring the spatial separation of the Ca^2+^ signals. However, despite this overlap, there was a clear difference in the regions showing a Ca^2+^ increase for each modality of stimulation, suggesting that the synaptic inputs to the dendrites of DBNi1-2 are separated according to modality and that this is reflected in localized increases in intracellular Ca^2+^ as demonstrated in Fig. 2*Aiv* and schematically represented in Fig. 2*C*. This also suggests that local synaptic inputs are driving the Ca^2+^ increase, and not spikes, which would be expected to drive a more widespread Ca^2+^ increase.

#### Time course of Ca^2+^ signals.

To assess the amplitude and time course of Ca^2+^ signals in different dendritic areas, separate ROIs were used to select the dendritic regions (Fig. 2*Di*, fr, jr, and ar). To quantify the relative increase in Ca^2+^ to each modality of stimulation, the change in fluorescence from baseline (ΔF/F) was measured in each ROI (Fig. 2, *Dii–Div* and *Ei*) and normalized to the maximum fluorescence in all ROIs to each modality of stimulation (Fig. 2*Eii*). In all ROIs, the greatest ΔF/F occurred in response to flagellar stimulation (Fig. 2, *Dii–Div* and *Ei*); however, scatter may have influenced this effect, as well as spike-derived Ca^2+^ signals. Without normalization, the response to touch receptor stimulation was 7.9 ± 2.8% and occurred in the fingerlike region (Fig. 2*Eii*). Antennal base stimulation induced the highest ΔF/F, 2.3 ± 0.1%, in the junction region (Fig. 2, *Dii–Div* and *Ei–Eii*). Visual stimulation induced the largest ΔF/F, 2.2 ± 0.5%, in the anterior region (Fig. 2, *Dii–Div* and *Ei–Eii*). Measured in the ROI with the highest ΔF/F, the decay of the Ca^2+^ response was similar for each stimulus modality, with a time constant (τ_d_) of 1.1 ± 0.4 s for flagellar stimulation, 1.2 ± 0.4 s for antennal base stimulation, and 1.4 ± 0.4 s for visual stimulation (τ_d_ to 37%, *P* = 0.54, ANOVA, *N* = 4).

#### Localization of Ca^2+^ signals upon flagellar stimulation.

**Fig. 3.**
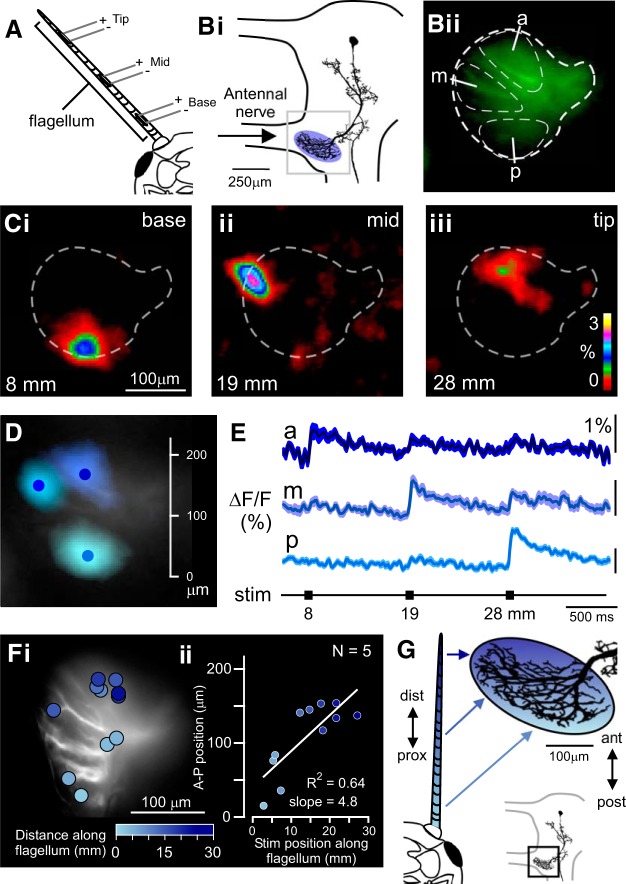
Topographic organization of Ca^2+^ increases for flagellar touch receptors. *A*: stimulating electrodes were inserted at 3 positions along the flagellum. *Bi*: imaging of the fingerlike dendrites. Outline of the brain shows the region of mechanosensory afferent input (blue oval) and the area imaged (gray square). *Bii*: mean of 50 frames of in vivo Ca^2+^ signal. The fingerlike dendritic arborization is outlined by a thick dashed line. Regions of interest (ROIs) covering the anterior (a), middle (m), and posterior (p) dendrites are indicated by thin dashed lines. *Ci–Ciii*: binned fluorescence increase (ΔF/F, %) in one animal on stimulation at 8 mm (5 repeats; *i*), 19 mm (5 repeats; *ii*), and 28 mm (3 repeats; *iii*), from the base of the flagellum. Data are binned over 5 pixels in *x* and *y*, and over 2.5 frames in time. *D*: overlay of pixels with mean peak ΔF/*F* >50% of the maximum response, with stimulation at 8 mm (light blue), 19 mm (blue), and 28 mm (dark blue) along the flagellum. Data are binned over 10 pixels in *x* and *y*, and over 5 frames in time. *E*: time course of fluorescence responses (ΔF/F, %) in the ROIs on stimulation (stim) at 8 mm (a), 19 mm (m), and 28 mm (p) from the base of the flagellum. ROIs are as indicated in *Bii*. *Fi*: midpoints of fluorescence responses on electrical stimulation at different points along the flagellum. The distance from stimulation sites to the base is indicated by shades of blue (color scale at *bottom*). *Fii*: correlation between the positions of electrical stimulation along the flagellum as measured from the base and the position of midpoint of fluorescence along the anteroposterior (A-P) spread of the dendrites. *G*: schematic diagram of topographic map as indicated by localized Ca^2+^ signals. Afferents from the flagellum project to the brain with their proximal (prox)-to-distal (dist) position represented along the posterior (post)-to-anterior (ant) axis of the neuropile. Shades of blue represent corresponding topography. Position of flagellar neuropile in the brain is indicated by a black box in *inset* diagram.

Because the midpoint of fluorescence differed between animals with their flagellum stimulated at various points (Fig. 2*Bi*), the correlation between the position of antennal stimulation and the localization of the fluorescence increase in the fingerlike region of the dendrites was explored. The total length of the flagellum varied from 20 to 28 mm between animals (mean: 25 ± 2 mm, *N* = 10 males and 10 females). Up to three extracellular stimulating electrodes were inserted into the flagellum at points between 2 and 28 mm from the base (Fig. 3*A*). Only the fingerlike dendrites of DBNi112 were imaged during these experiments (Fig. 3*B*).

The fluorescence profile in DBNi1-2 is presented for a single animal with three stimulating electrodes inserted into its flagellum (Fig. 3*C*). Stimulation at 8 mm from the base of the scape elicited the highest ΔF/F in a posterior region of the fingerlike dendrites, with the midpoint 109 μm medially from the lateralmost point and 40 μm anteriorly from the posteriormost point of these dendrites (Fig. 3C*i* and Supplemental Movie S2). Stimulation at the middle of the flagellum (19 mm from the base) elicited the highest ΔF/F in a region more lateral and anterior, with the midpoint 37 μm medially and 121 μm anteriorly (Fig. 3C*ii*). Stimulation close to the tip (28 mm from the base) induced the highest ΔF/F in a more medial and anterior region, with its midpoint 112 μm medially and 142 μm anteriorly (Fig. 3C*iii*). When overlaid, these regions of highest ΔF/F were clearly separated (Fig. 3*D*). In addition, when ROIs were used to select an anterior, middle, and posterior region of the fingerlike dendrites (Fig. 3B*ii*), an independent fluorescence increase occurred in each ROI for the different positions of flagellar stimulation, with very little cross talk (Fig. 3*E*). The amplitude of the Ca^2+^ increase differed between each region.

When averaged between 11 stimulation positions in 5 animals, a positive correlation was found between the position of the stimulating electrodes along the flagellum and the position of the midpoint of fluorescence (Fig. 3*Fi*): the anterior distance of the midpoint scaled linearly with a slope of 4.8 μm from the posterior tip of the dendritic arborization per millimeter distance along the flagellum (Fig. 3*Fii*; *R*^2^ = 0.64, *P* = 0.003, *N* = 5). Together, these data suggest that a topographic map of touch to the flagellum exists in this antennal neuropile region, with the proximal-to-distal axis of the flagellum represented along the posterior-to-anterior axis of the neuropil and the DBNi1-2 fingerlike dendrites (summarized in Fig. 3*G*).

The topography of inputs from the visual system and the campaniform sensilla was not investigated. The campaniform sensilla come in a small cluster at the base of the antenna (Fudalewicz-Niemczyk and Rościszewska 1973; Staudacher et al. 2005) and only form synapses with a restricted area of DBNi1-2 (Schöneich 2008).

#### Relationship between Ca^2+^ signals and spiking.

**Fig. 4.**
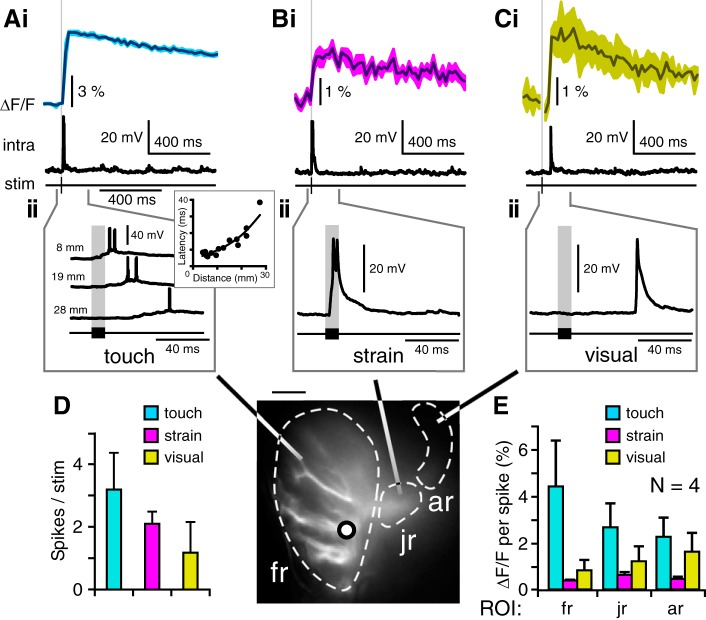
Relationship between Ca^2+^ signals and spiking response. *A–C*: Ca^2+^ signal (ΔF/F, %) in regions of interest (ROIs; fr, fingerlike dendrites; jr, junction region; ar, anterior region) are aligned with intracellular recording from a single branch of the fingerlike dendrite. ROIs (stippled lines) and recording site (black circle) are indicated in image at *bottom center*. Data are from the same animal; Ca^2+^ signal and electrophysiology were recorded subsequently as microelectrode was removed to use high-power objective. *Ai–Ci*: responses of fr to touch receptor stimulation (*Ai*), strain receptor stimulation (*Bi*), and visual stimulation (*Ci*). Stimulation times (stim) are indicated by gray line and black bar below. *Aii*: spike response to touch receptor stimulation at higher temporal resolution. Traces are shown for 3 positions of electrical stimulation along the flagellum, as indicated at *left*. A plot of latency to first spike against distance along the flagellum is given in *inset*. *Bii*: intracellular recorded response to strain at higher temporal resolution. *Cii*: response to visual stimulation. *D*: quantification of the intracellular response with spikes per stimulus. *E*: quantification of imaging response ΔF/F (%) per spike (*N* = 4) for each ROI (fr, jr, and ar) and for each mode of stimulation (touch, strain, and visual stimulation).

Because Ca^2+^ indicators were introduced with intracellular microelectrodes, for the same stimuli the spiking response of DBNi112 could be compared with the fluorescence signals. The electrode was usually inserted into one of the large branches of the fingerlike dendrites (fr in Fig. 4, *bottom center*). Stimulating touch-sensitive receptors in the middle of the flagellum (14 ± 1.8 mm) elicited 2.2 ± 0.6 spikes (*N* = 4; Fig. 4*Aii*). The latency to the first spike varied, from 6.4 to 38.5 ms, depending on the distance of the stimulation site from the antennal base (*N* = 7; Fig. 4*Aii*, *inset*). Stimulation of strain receptors at the antennal base elicited 2.5 ± 0.5 spikes at a latency of 5.5 ± 0.4 ms (*N* = 7; Fig. 4, *B* and *D*), and visual stimulation elicited 1.4 ± 0.3 spikes at a much longer latency of 53 ± 1.8 ms (*N* = 7; Fig. 4, *C* and *D*). In each case, a rapid Ca^2+^ increase occurred concurrently with the onset of spiking, increasing to its maximum intensity within 40 ms, or 2 frames of imaging (Fig. 4, *Ai–Ci*). The decay in fluorescence was much slower and took over 1 s, as noted previously.

In three ROIs (fr, jr, and ar in Fig. 4, *bottom center*), the relative ΔF/F was compared within each animal with the number of spikes to each stimulus (Fig. 4*E*). The ΔF/F occurring with a spike was different for each modality of stimulation: for example, in the fingerlike area, flagellar stimulation induced the highest increase, 4.4 ± 2.0% ΔF/F per spike, whereas in the junction area, strain to the antennal base induced 0.6 ± 0.1% ΔF/F per spike, and in the anterior area, visual stimulation induced 1.6 ± 0.8% ΔF/F per spike (Fig. 4*E*). The latter value is likely to underestimate ΔF/F to visual stimulation, because the anterior dendrites receiving visual input are located deep in the brain. The range of ΔF/F coupled with single spikes provides further indication that the fluorescence signal in the dendrites was not derived from spike activity.

#### Effect of Ca^2+^on multimodal processing.

To test the effects of Ca^2+^ within DBNi1-2, Cd^2+^, a Ca^2+^ channel blocker, was bath-applied to the preparation. Bath application should not affect spike generation in afferent neurons because they are located peripherally, in the antennae or optic stalk.

**Fig. 5.**
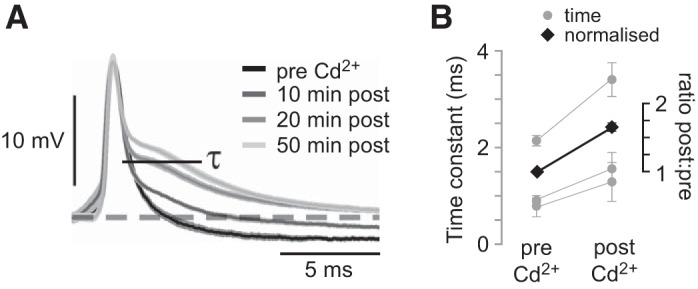
Effect of Cd^2+^ on spikes generated by DBNi1-2. *A*: action potentials recorded intracellularly before and after application of Cd^2+^. Values are means ± SD of 5 spikes at each time before (pre) and after (post) application. The time constant (τ) of the repolarization phase was measured for all spikes. *B*: mean values of τ for 20 spikes before and 20 min after bath application of Cd^2+^ are indicated by circles (time, *left* axis; data are from 3 different preparations). Relative value of τ normalized to response before application of Cd^2+^ is represented by diamonds and pooled over 3 experiments (normalized post:pre, *right* axis).

Intracellular recordings of spikes of DBNi1-2 were compared before and after application of Cd^2+^. Individual spikes occurring with a large interspike interval and generated without direct sensory simulation were used to avoid analyzing compound responses. The duration of the K^+^-dependent repolarization phase of each action potential increased gradually over time (Fig. 5, *A* and *B*). Twenty minutes after application of Cd^2+^, an overall mean significant increase in the time constant τ of the repolarization phase occurred, from 1.3 ± 0.4 to 2.1 ± 0.7 ms (Fig. 5*B*; *P* = 0.04, paired *t*-test, *N* = 3). This change in action potential shape indicates that a reduction or absence of Ca^2+^ in the neuron results in fewer K^+^ channels being open, suggesting that Ca^2+^-sensitive K^+^ currents are present (Savić et al. 2001; Sivaramakrishnan and Oliver 2001). Combined with the discovery of localized Ca^2+^ currents in DBNi1-2, this provides a possibility that Ca^2+^ is directly involved in multimodal processing through localized changes in K^+^ ion conductivity.

## DISCUSSION

We have investigated how the dendritic structure of a cricket antennal neuron, DBNi1-2, relates to the processing of three modalities of sensory input: touch, strain, and visual stimulation. Spatially restricted increases in Ca^2+^ suggest that the sites of synaptic input to the neuron are different for each modality (Fig. 2 and Supplemental Movie S1). Ca^2+^ signals were further divided for the inputs from touch receptors along the flagellum, which appear to be arranged in a topographic map (Fig. 3 and Supplemental Movie S2). Localized Ca^2+^ increases in DBNi1-2 may induce Ca^2+^-sensitive K^+^ currents (Fig. 4), as tested by applying Cd^2+^ to the preparation (Fig. 5).

### 

#### Separation of synaptic inputs.

For all sensory modalities tested, the localized Ca^2+^ increases recorded (Fig. 2) indicate a close correspondence between the morphological projection sites of the relevant sensory neurons described in previous studies (see Introduction) and the location of synaptic input onto the dendrites of DBNi1-2.

Electrical stimulation to the flagellum drove a Ca^2+^ increase in the fingerlike dendrites of DBNi1-2, in agreement with their morphological overlap with mechanosensory flagellar afferent fibers (Staudacher and Schildberger 2000). By stimulating the afferent fibers at different lengths along the flagellum, we revealed a topographic map of flagellar afferent projection, with basal afferents projecting posteriorly onto DBNi1-2 and distal afferents projecting anteriorly (Fig. 3 and Supplemental Movie S2). Topographically arranged Ca^2+^ signals have previously been observed in the auditory neurons of crickets (Baden and Hedwig 2007), and bush crickets (Prešern et al. 2015), as well as in other insect neurons that receive inputs from maps of different sensory modalities, including neurons that respond to wind stimulation of the cerci of crickets (Ogawa et al. 2004) and to visual stimulation of the eyes of flies (Borst and Egelhaaf 1992; Spalthoff et al. 2010) and locusts (Peron et al. 2009).

When the antennal base was stimulated mechanically, the region of highest fluorescence increase was at the junction region of the fingerlike dendrites of DBNi1-2 (Fig. 2, *Aii* and *Aiv*), where the campaniform sensilla synapse onto the neuron (Schöneich 2008). However, a small fluorescence increase was also observed in a posterior region of the fingerlike projections (Fig. 2, *Aii* and *Aiv*). This is likely to be due to simultaneous activation of the trichoid sensilla on the basal flagellum, which project to the posterior dendrites according to the topographic arrangement (Fig. 3).

Frontal visual input to the compound eyes led to a midpoint of fluorescence increase in DBNi1-2 that was specific to an anteriorly located dendrite (Fig. 2, *Aiii*, *Biii*, *D*, and *E*), suggesting that the neuron receives visual input within this region. Although this anterior dendrite was located too deep within the brain to be imaged optimally with our system, no other dendritic region responded to the visual stimulus. A two-photon microscope would allow deeper imaging with a higher spatial resolution (Helmchen and Denk 2005).

In insects, stimulus-specific local Ca^2+^ signals have also been observed in the locust lobula giant movement detector (Peron and Gabbiani 2009; Peron et al. 2009), cercal giant interneurons in the cricket (Ogawa et al. 2004, 2008), and auditory neurons in the cricket (Baden and Hedwig 2007; Sobel and Tank 1994) and bush cricket (Prešern et al. 2015; Triblehorn and Schul 2013). Previous work investigated responses to a single stimulus modality, whereas we studied the response to multiple modalities within a single neuron.

#### Ca^2+^ localization based on stimulus type.

The localization of Ca^2+^ signals to specific dendritic regions implies that downstream effects of Ca^2+^ should also occur locally. The influence of Ca^2+^ on spike shape in DBNi1-2 was tested with Cd^2+^, which is known to block voltage-sensitive Ca^2+^ channels (Tang et al. 2014) and to reduce Ca^2+^ influx on activation of nicotinic acetylcholine receptors (Thany et al. 2008). The mechanosensory input to DBNi1-2 is likely cholinergic, similar to locust antennal afferents (Knipper et al. 1989). Therefore, Cd^2+^ should block any Ca^2+^ influx from their activation. In addition, Cd^2+^ may have a wide range of effects in neurons. For example, in presynaptic terminals, Cd^2+^ reduces Ca^2+^ influx (Heidelberger and Matthews 1992; Mintz et al. 1995) and synaptic efficacy (Mintz et al. 1995). Twenty minutes after application of Cd^2^, the K^+^-dependent repolarization stage of action potentials was elongated (Fig. 5, *A* and *B*). This indicates that Ca^2+^ in DBNi1-2 affects K^+^ currents, which are reduced when Ca^2+^ is reduced or absent after addition of Cd^2+^. In future work, this could be corroborated, such as with the use of BAPTA (Prešern et al. 2015; Triblehorn and Schul 2013). The basis for this effect could also be studied by focusing on Ca^2+^-sensitive K^+^ channels, cAMP signaling, or channel phosphorylation (Wicher et al. 2001). The use of pharmacological agents specific to particular classes of Ca^2+^ channels should shed further light on the role of Ca^2+^ in the neuron. However, many agents effective in vertebrates, such as apamin, are not effective in insects (Wicher et al. 2001), making the identification of these channels currently more difficult.

The separation of sensory inputs has important functional implications for multimodal processing (reviews: Koch and Segev 2000; London and Häusser 2005). For example, the passive electrotonic spread of synaptic potentials in one dendritic region may have little effect in distant regions (Grimes et al. 2010; Jacobs et al. 1986; Liu 2004; Polsky et al. 2004). Dynamic properties may also contribute to localized synaptic processing, such as activity-dependent adaptation (Sanchez-Vives et al. 2000; Savić et al. 2001; Triblehorn and Schul 2013) or long-term weakening or strengthening of synapses (review: Larkum and Nevian 2008). Assessing the extent to which these occur within DBNi1-2 would be an interesting avenue for future research.

The localization of ion channels also has important functional implications: if Ca^2+^-sensitive K^+^ currents occur in the dendrites, their activation could lead to a reduction in response to a subset of inputs (Kurtz et al. 2000; Prešern et al. 2015); however if present close to the spike-generating zone, it could lead to a reduction in the response to all stimuli (Benda and Herz 2003; Peron and Gabbiani 2009). Higher resolution optical imaging of single dendrites could provide more details of the localization of Ca^2+^ currents.

#### Role of DBNi1-2 in behavior.

The functional role of DBNi1-2 in behavior is not yet known. Its projections to the thoracic ganglia are mapped (Schöneich et al. 2011), and a similar neuron, DBNi2-1, has been implicated in turning behavior when depolarized (Zorović and Hedwig 2013). DBNi1-2 also shows morphological similarities to a giant neuron in the cockroach (Comer and Baba 2011) that has also been implicated in turning (Ye and Comer 1996). Multimodal processing could therefore be important to influence the decision to turn.

#### Relevance to other systems.

This work was undertaken on an insect neuron, which provides two major advantages: first, it is easily accessible for intracellular recordings, and second, it overlaps with large areas of a sensory neuropile, allowing the nature of synaptic inputs to each of its dendritic regions to be functionally mapped. These fundamental properties of DBNi1-2 are comparable to those of neurons in the mammalian nervous system. In a wide range of neurons, functionally different inputs synapse onto different dendritic regions, the location of which can be estimated by using Ca^2+^ imaging (amacrine cells: Euler et al. 2002; Grimes et al. 2010; cortical pyramidal neurons: Hill et al. 2013; hippocampal pyramidal neurons: Sheffield and Dombeck 2015; cerebellar Purkinje cells: Kitamura and Häusser 2011). The compartmentalization of Ca^2+^ signaling allows for local computation through a range of downstream effects (reviews: Branco and Häusser 2010; London and Häusser 2005) and demonstrates the complexity of the processing in single neurons.

## GRANTS

This work was supported by a PhD grant from the Medical Research Council UK (to T. G. Bayley).

## DISCLOSURES

No conflicts of interest, financial or otherwise, are declared by the authors.

## AUTHOR CONTRIBUTIONS

T.G.B. and B.H. conceived and designed research; T.G.B. performed experiments; T.G.B. analyzed data; T.G.B. and B.H. interpreted results of experiments; T.G.B. prepared figures; T.G.B. drafted manuscript; B.H. edited and revised manuscript; T.G.B. and B.H. approved final version of manuscript.

## Supplemental Data

Movie 1 Movie 1: Ca^2+^ response of DBNi1-2 to stimulation of different modalities, in real time. (.mp4 3 MB)

Movie 2 Movie 2: Ca^2+^ response of DBNi1-2 to electrical stimulation at different points along the flagellum. (.mp4 790 KB)

Movie Legends Legends for Movies 1 and 2(.docx 12 KB)
