# Supplementary material for: Dendritic Ca2+ dynamics and multimodal processing in a cricket antennal interneuron
Source: J Neurophysiol. 2018 May 9;120(3):910–9. doi: 10.1152/jn.00663.2017 (PMC6171068; doi:10.1152/jn.00663.2017)
Supplement: Movie Legends — Legends for Movies 1 and 2(.docx 12 KB) [file movie-legends.docx]

# Movie legends

**Movie 1:** Ca^2+^ response of DBNi1-2 to stimulation of different modalities, in real time. Raw Ca^2+^ signal on left, ΔF/F on right, and merge of raw signal and ΔF/F in centre. Response shown for electrical stimulation with two wire electrodes inserted into the flagellum (flagellum, cyan), tactile stimulation with a pin on the base of the antenna (base, magenta), and visual stimulation with an LED in front of the animal (visual, yellow). Each stimulus is 10 ms long. First sequence shows response to the three stimulation types presented sequentially; recorded with a 10x dipping lens at 50 Hz. Open circle represents stimulus last presented, closed circle represents presentation of stimulus. Second sequence shows response to flagellar stimulation; recorded with a 20x dipping lens at 50 Hz. Third sequence shows response to antennal base stimulation; recorded with a 20x dipping lens at 50 Hz. Fourth section shows response to visual stimulation; recorded with a 10x dipping lens at 20 Hz. Time from presentation of first stimulus is given in top left, in seconds. Colour bar indicates ΔF/F. Colour scale differs between sections. First section: black – 0 %, yellow – 10 %; second section: black – 0 %, yellow – 12 %; third section: black – 0 %, yellow – 2 %; fourth section: black – 0 %, yellow – 3 %. Mean of at least 10 presentations per stimulus. Scale bar – 100 μm.

**Movie 2:** Ca^2+^ response of DBNi1-2 to electrical stimulation at different points along the flagellum. Raw Ca^2+^ signal on left, ΔF/F on right, and merge of raw signal and ΔF/F in centre. Three pairs of wire electrodes were inserted into the flagellum at different points: 8mm, 19 mm, and 28 mm from the base of the scape. Open circle represents position of stimulus last presented, closed circle represents presentation of stimulus. A Ca^2+^ signals occurs in different dendritic regions for each stimulus position. Time from presentation of each stimulus is given in top left, in seconds. Colour scale indicates ΔF/F: black – 0 %, yellow – 3 %. Mean of 3-5 presentations per stimulus. Scale bar – 100 μm.
